# Transforming Nursing Education to Strengthen Health System in Malawi: An Exploratory Study

**DOI:** 10.2174/1874434601812010093

**Published:** 2018-05-31

**Authors:** Thokozani Bvumbwe, Ntombifikile Gloria Mtshali

**Affiliations:** 1 Faculty of Health Sciences, Mzuzu University, Mzuzu, Malawi; 2 School of Nursing and Public Health, University of KwaZulu Natal, KwaZulu Natal, South Africa

**Keywords:** Malawi, Nursing education, Quality, Quantity, Relevance, Strategies, Tranformng, Scale up

## Abstract

**Background:**

Malawi made great strides to increase the number of nurses through the Emergency Human Resource for Health Program. However, quantity of health workforce alone is not adequate to strengthen the health system. Malawi still reports skill mix imbalance and geographical mal-distribution of the nursing workforce. Health systems must continuously adapt and evolve according to the health care needs and inform health professionals’ education to accelerate gains in health outcomes. The Lancet Commission reported that health professionals’ education has generally not lived up pace with health care demands.

**Objectives:**

The aim of this study was to describe the strategies being implemented in Malawi to improve nursing education. Specifically, the objectives of the study were to explore strategies being implemented, identify stakeholders and their targets in order to share practices with countries experiencing similar nursing education challenges.

**Methods:**

This was a cross sectional descriptive study with a concurrent mixed method design. One hundred and sixty participants including nurse practitioners and educators responded to a questionnaire. Fifteen nurse practitioners and eight nurse educators were also engaged in one to one interview.

**Results:**

Respondents showed varied opinion on how nursing education is being implemented. Six themes as regards strategies being implemented to improve nursing education emerged namely- capacity building, competency based curriculum, regulation, clinical learning environment, transformative teaching and infrastructure/ resources.

**Conclusion:**

Findings of this study show that the strategies being implemented to improve nursing education are relevant to closing the gap between health care needs and nursing education.

## 1 INTRODUCTION

A competent nursing workforce is a critical building block for an effective health system [1]. Nurses form the backbone of the health system and are a universal access point for almost 90% of healthcare users in Sub Saharan Africa [2-4]. However, literature reports severe shortage of nurses in the region. Malawi has a nurse based health system but still has a vacancy rate of 65% for nursing and midwifery positions in public sector [5]; and a geographic mal-distribution of 74% of providers in urban serving 19% of the population [6]. Malawi requires 20% more nurses to meet healthcare needs [6].

The World Health Organization through a resolution at the World Health Assembly 59.27 urged member states to strengthen nursing and midwifery by establishing comprehensive programs to train nurses and midwives [7]. The Plan of Action for Scaling up Quality Nursing and Midwifery Education and Practice for the African Region 2012 – 2022, Transforming and scale up heath professionals’ education and training guidelines, the Regional Professional Regulatory Framework for Nursing and Midwifery, the Four Year integrated nursing and midwifery competency- based prototype curriculum for the African Region provide adequate framework for improving quantity, quality and relevance of nurses and midwives at region level. The National Nurse Scale up Operational Plan highlighted need to double nursing intake to solve the human resource for health crisis in Malawi [8].

Between 2004 – 2010, Malawi greatly increased the number of nurses through the Emergency Human Resource for Health Program [5]. However, quantity of health workforce alone is not adequate to strengthen the health system. Malawi still reports skill mix imbalance [9] and geographical mal-distribution of the workforce [10]. To accelerate gains in health outcomes, health systems must continuously adapt and evolve according to the health care needs. The changing health care landscape should inform changes in the health care professionals’ education system. However, health professionals’ education has generally not coped up with health care needs and demands [11] and competencies of health workers do not match health needs. There is poor collaboration among professionals. Professional education is still hospital based care at the expense of community based care.

## 2 AIM

The purpose of this study was to describe strategies being implemented in Malawi to improve nursing education. Specifically, the objectives of the study were to explore strategies being implemented, identify stakeholders and their targets in order to share practices with countries experiencing similar nursing education challenges.

## 3 MATERIALS AND METHODS

### 3.1 Design

This was a cross sectional descriptive study done as part of a doctorate study to analyze strategies aimed at improving quality, quantity and relevance of nursing education in Malawi. A concurrent mixed method design involving both quantitative and qualitative approaches was used to describe strategies being implemented.

### 3.2 Participants and Data Collection

Participants of this study were nurse practitioners from three teaching hospitals and nurse educators from four nursing educational institutions. The hospitals and the nursing educational institutions were systematically sampled from sampling frames of 10 teaching hospitals and 19 nursing educational institutions respectively. For the hospitals, every third hospital name was picked. Similarly every fourth training institution was picked on training institutions sampling frame. Data were collected from June 2015 – December 2015. The study utilized the Roasoft sample size calculator with a margin error at 5%, confidence interval at 95% and a total population of 269 nurses to engage a minimum recommended sample size of 159. Researchers distributed questionnaires to nurse practitioners who were found on daily basis for a period of one week at each sampled institutions. A pilot study that involved three nurse educators and five nurse practitioners from a college and a hospital not included in the study sampled helped to refine the questionnaire.

Data were collected using self-administered questionnaires and face-to-face interviews. The questionnaire consisted of a demographic characteristics section and Likert scaled items on four major areas of nurse training including; student recruitment, teaching and learning, clinical practice and transition to practice. Participants were requested to give their rating on aspect of each of the items in the questionnaire were been implemented. A Likert scale with the following options -strongly agree, agree, neutral, disagree, and strongly disagree was used.

Fifteen nurse practitioners and eight nurse educators were then purposively engaged in one face-to-face interview to describe the strategies being implemented to improve nursing education at their institutions. The nurse practitioners were also those trained as clinical preceptors because they had specific knowledge of strategies being implemented to improve nursing education in Malawi. An unstructured interview guide with probes was used. The guide included questions to solicit participants’ knowledge and experiences of strategies being implemented to improve nursing education,

### 3.3 Data Analysis

Completed questionnaires were coded and entered onto Statistical Package for the Social Sciences (*SPSS* v16.0). Descriptive statistics using Means and standard deviations were analysed.

For the interviews, content analysis, a systematic coding and categorizing approach was used to analyse qualitative data [12]. The researcher listened to the interviews carefully and data was transcribed verbatim. Transcribed data was read repeatedly to gain a deeper sense of the content. Careful and repeated reading of transcribed text helped to identify patterns and trends of participants’ responses. The researcher identified meaningful words from the transcripts. Codes were used to categorize responses based on their similarities and differences.

### 3.4 Ethics

Ethical approval was obtained from the University of KwaZulu Natal (HSS/0986/012D) and Malawi Ministry of Health (NHSRC # 1154). Permission was also sought from hospital directors and college principals. Respondents signed a written informed consent before participating in the study. They were free to withdraw from the study at any time. Data was kept anonymous as only codes were used on the questionnaires. Participants were informed that there was no perceived risk for participating in the study.

### 3.5 Academic Rigor

The questionnaire was pre-tested with ten nurse practitioners and five educators to ensure that the questionnaire could easily be responded to by participants and that it would solicit the appropriate responses. Questions that were not clear were reviewed and updated. The trustworthiness of the qualitative data was ensured using two approaches. Firstly a “member checking” method was used. The researcher contacted at least three of nurse practitioners and three nurse educators to verify and confirm the authenticity of the results. The researcher shared the summary of data analysis and results to check whether the developed concepts really reflect the situations and ideas that were discussed during interviews [13]. Secondly “peer checking” was used to check the credibility of the data analysis. Two fellow members of staff from the researcher’s educational institution were requested to independently identify concepts and codes from the data. These were compared with the researchers’ developed codes and categories. Differences were adjusted and harmonized through a discussion.

## 4 RESULTS

### 4.1 Demographic Characteristics

The study distributed a total of one hundred and sixty eight questionnaires with a 95% (n=160) return rate. Findings of the study showed that 76% (n=121) of the respondents were nurse practitioners and 25% (n=39) were nurse educators. Eighty five percent (n=136) were female and 15% (n=24) were males. Respondents’ age ranged from 21 to 45 years with a median age of 28 years. About 56% (n=90) were college diploma (NMT) holders, 29% (n=46) diploma (RN) holders, 11% (n=18) bachelor degree and 4% (n=6) were master degree holders (Table **1**).

### 4.2 Views on Nursing Education in Malawi

Participants were engaged to rate their opinions on aspects of nursing education in Malawi. A self-developed questionnaire was used for participants to rate their views as regards students’ recruitment into nursing programs, teaching and learning, clinical practice, and transition to practice (Table **2**). The results indicated that there were proper systems for recruiting students into nursing programs (mean=1.30, SD=0.77), standards for recruiting students are in place (mean=1.06, SD=0.24). However, participants felt that they have not come across any strategies to improve student recruitment (mean=4.56, SD=0.68). Most members strongly agreed with items under teaching and learning. Findings show that participants noted availability of strategies to improve clinical practice (mean=1.57, SD=0.71). Participants indicated that there is slightly poor collaboration between faculty and clinical personnel (mean=2.62, SD=1.10).

Participants disagreed to most items within transition to practice setting. Participants felt that there is no formal transition program in place for newly graduated nurses (mean=3.61, SD=1.17). Findings show that nursing educators are not involved in transition of graduates to practice (mean=4.39, SD=0.74), there are no transition to practice guidelines (mean=4.69, SD=0.53) nor are there strategies to improve transition to practice among nurses.

### 4.3 Nursing Education Stakeholders

There are a number stakeholders working on improving nursing education in Malawi (Table **3**). Findings show that there is collaboration among stakeholders because they all launch their programs through the ministry of health. The overall goal of stakeholders’ programs was to strengthen the health system. The strategies being implemented are common among colleges.

### 4.4 Transforming and Scale up Strategies for Nursing Education

Six themes emerged from the analysis of the excerpts. These were (i) capacity building, (ii) competency-based curriculum (iii) clinical learning environment (iv) regulation (v) transformative teaching and (vi) infrastructure/resources. Table **4** summarizes the strategies being implemented and the strategies’ specific area of focus.

#### 4.4.1 Themes 1: Capacity Building

There are more nurses who have undergone graduate studies with support from identified stakeholders to improve nursing. Nursing education and practice stakeholders have prioritized faculty development as a key strategy.

“...There are more opportunities for graduate studies than it was years back due to plans to increased nursing faculty to master level..”.

Educational institutions have doubled intakes as a strategy to solve the nursing shortage. However, the shortage of full time faculty frustrates the efforts. Educational institutions have embarked on employing adjunct faculty both for classroom and clinical teaching. In most cases, adjunct staff come from practice setting. Participants of the study reported that capacity building programs for adjunct staff include mentorship, teaching methods and clinical assessment methods.

“..A good number of faculty members are in training and we involve adjunct staff from clinical area. We orient them in teaching methods ”

Students in all nursing programs rotate through blocks of theory and clinical practice. However, because of shortage of nursing faculty, educational institutions are dependent on the clinical nurses in the hospital to teach students. Participants reported that preceptorship has been adopted to build capacity of clinical nurses to manage students during clinical learning. Preceptorship, mentorship and clinical teaching programs were developed to equip registered nurses with knowledge and skills to handle students. One hundred twenty nine preceptors across the country have been trained with funding from ICAP and Clinton Health Initiative (CHAI). Nurse midwife technicians (NMTs) are in majority in Malawian hospitals than registered nurses. Students interact with NMTs more often than they do with registered nurses. A program to orient NMTS to clinical teaching is also in place.

“...Nurse midwife technicians teach our students and they have to know how on managing students during clinical allocation.”

#### 4.4.2 Theme 2: Competency Based Curriculum

Nurses and Midwives Council of Malawi (NMCM) determines syllabus for nursing programs. Syllabus development takes into account the prevailing health care environment identified within national health policies and frameworks. Participants reported that NMCM has taken every effort to review and align the curriculum to meet national and international standards. However, participants reported that curriculums are still hospital based on the expense of primary health care.

“..The minimum hours for nursing training prioritize curative other than promotive and preventive services”.

There is need to adopt a more community based education in order to revitalize primary health care for universal coverage.

“…Students should have adequate time to practice in the community..”.

Findings of the study show that inter-professional education is not well established in Malawi. Participants highlighted that usually students from different profession meet informally during clinical practice. There are no formal programs that are encouraging inter-professional training apart from a doctorate degree program offered by one University College. The healthcare system is demanding a wide range of knowledge and skills for nurses to efficiently and effectively manage the comprehensive needs of patients and populations.

Competency based curriculum has been adopted in most educational institutions. Competency based education describes progression through training. Students demonstrate ability to perform certain tasks. Competence-based curriculum ensures a relevant workforce that would be responsive to health needs because a set of defined competencies are derived from the health system landscape and demands.

“..The new approach forces educators to focus on individual skills. Student is given the necessary attention”

“.Competency based makes students ready for work..”

#### 4.4.3 Theme 3: Clinical Learning Environment

The clinical learning environment is with more challenges. Shortage of registered nurses to teach students and lack of learning resources were reported. Lack of faculty support to clinical nurses was identified as a cause of poor attitude of nurses towards students, ineffective clinical assessments and poor attitude of students towards patients. However, findings of the study reported innovative approaches to ensure effective clinical learning environment. NEPI established clinical model teaching wards at selected hospitals. The idea of the teaching wards is to provide students with an ideal clinical learning environment with adequate resources and trained preceptors.

“…The model wards have the necessary procedure manuals, practice guidelines which are not there in non-model wards”.

There is an overcrowding of students. Participants indicated that much as educational institutions have doubled their intakes, clinical learning sites remained the same. This necessitated establishment of clinical audit teams to ensure coordinated clinical allocations.

“ ..Colleges jointly plan student placement to avoid congestion”.

#### 4.4.4 Themes 4: Regulation

A quest for educational institutions that train NMTs to partner with universities in order to develop and offer pre-service programs at registered nurse level, is also growing. Consequently, the Nurses and Midwives Council of Malawi regulation demands a university bachelor’s degree as a minimum qualification to teach registered nurses. Increasing number of educational institutions to train nurses at registered nurse level will improve service delivery. Registered nurses are equipped with analytical and critical thinking skills.

“.. Our college is developing a registered nurse program. This will need our lecturers to have a minimum of master degree.

NMCM introduced accreditation system for educational institutions. Accreditation system facilitates educational institutions to work towards acceptable standards. The council also conduct supportive supervision to educational institutions. Nursing education should strive to maintain quality of its graduates in order to guarantee public safety.

“ ..All colleges are expected to be accredited for them to be licenced to train nursing. This controls quality”.

NMCM also regulate content to be taught at various levels and programs. Participants highlighted that the scope of practice is relevant for each cadre. There has been capacity building of various committees of the council in areas like quality assurance, monitoring and evaluation, testing and measurements.

#### 4.4.5 Themes 5: Transformative Teaching

Doubling of student intake has brought its own challenges for teaching and learning. Findings show that there is overcrowding of students in the clinical area and often the students compete for resources and patients. Simulation is being used to imitate the clinical environment within the skills laboratory. Participants indicated that simulation is very significant because it exposes students to opportunities to care for conditions they could normally not find in the clinical setting.

E-learning has been attempted though appears to meet resistance.

“.. People are used to traditional approach of teaching and learning. One organisation is struggling to initiate e-learning because of resistance in some individuals”.

Participants noted that E-learning can be a best platform to help students’ access electronic resources. However, participants reported that lack of electronic resources has been the main challenge for e-learning in Malawi. Other participants noted that a number of students own a smart phone and this could be explored as to how the smart phones could be used for teaching and learning.

The Nurses and Midwives Council of Malawi introduced internship for graduates though this has not been implemented. Participants indicated that as students leave the training colleges they need more support to adequately integrate into practice setting. Internship would be an effective approach to ensure that graduates’ competencies are being strengthened before recruited into practice.

“..New graduates are expected to fully operate as competent nurses before they are effectively supported to be competent”

“..New graduates joining the system are expected to start work flat out. It’s a new environment and they need support.”

Innovations in students’ recruitment will be needed. Targeted recruitment from areas that are underserved might reduce maldistribution of nurses and midwives. Stakeholders have provided scholarships to needy deserving students. The Ministry of Health bonds the students to serve in public service for equivalent number of year of training. However, majority of participants noted that mechanisms to re-inforce the policy are inadequate.

“…We need measures to reinforce scholarship bonds because it is the only way we will ensure availability of nurses in underserved areas.”

#### 4.4.6 Themes 6: Infrastructure and Resources

Growing number of students has resulted in increased demand for more resources and infrastructure. Stakeholders have provided increased space for skills laboratory, student accommodation and libraries in some educational institutions. However, there is still demand for clinical consumables at both clinical settings and clinical skills laboratory. A cost sharing approach has been introduced. Educational institutions provide students with clinical consumables like gloves and basic clinical equipment like thermometers, stethoscopes and sphygmomanometer.

“.. We provide basic resources to students when they are going to the ward for clinical like gloves, apron, and thermometers”.

“When students go to the clinical area without the agreed items they are sent back because we agreed with hospitals to provide them with basic items”.

Participants also reported that educational institutions have embarked on an off campus accommodation approach. This has increased cost sharing between educational institutions and students.

“..We no longer are responsible for student’s accommodation. Students have to find and pay for their own accommodation and food. We are only responsible for their learning”.

Library books are becoming scarce among students. Access to electronic resources could improve access to books and journal for both faculty and students. However, intermittent availability of internet access is a challenge at most educational institutions. Computer literacy among students is not clear and needs to be investigated.

“..Books are expensive and college are failing to keep stock of adequate books”.

## 5 DISCUSSION

The purpose of this study was to describe strategies that are being implemented in Malawi to improve quality and relevance of nursing education. The Lancet Commission reported a gap between health needs and educational outcomes [15]. Many educational programs do not adequately address the needs of health care system [16]. While previous clinical teaching has traditionally focused on how and where clinical learning should take place, which clinical activities are required, how many hours should be acquired, improvements in clinical teaching and learning have focused on producing intended students learning outcomes [17]. Learning objectives often focus on what the leaner should know. Curriculum reform is central to transform the quality and relevance of nursing education. The recent focus on competency based curriculum call for identification of critical nursing activities and general competencies [18]. Competency based learning strengthens individual-based outcomes for each student. Transformative education demands that curricula be responsive to the needs of the society especially the neglected and marginalize poor populations [19].

As nursing education transformation is taking shape, findings indicated that inter-professional education is not well established in nursing education. However, inter-professional education is becoming a tool to add relevance to health professionals’ education worldwide [20]. Inter-professional education encourages members of more than one health profession to learn interactively together for the explicit purpose of improving inter-professional collaboration and overall health of the populations. Inter-professional collaboration has been linked to a range of outcomes, including improvements in patient safety and case management, the optimal use of skills of each team member and provisions of better health services [21]. With growing task shifting within the health care system [22], inter-professional education will enhance collaboration among healthcare teams [23].

Improving educational capacity through nursing faculty development has been pursued as one of the several strategies to address a complex human resource problem [24]. Steinert [25] described faculty development as “scholarly activities critical for promoting educational leadership, innovation, excellence and they are key to academic vitality”. Nursing education has witnessed a paradigm shift from traditional to emerging active, student- centered, transformative approaches to learning. Strategies to foster faculty development are significant because nurse educators are expected to do much more. Faculty development activities need to include opportunities for nurse educators to address and reshape norms to facilitate a successful transformation to a new educational paradigm [26].

NMCM reinforces mandatory continuous professional development for all nurses including faculty as a criteria to renew professional license. This is necessary considering that nursing faulty lose their practice competence over time. Nurse educators execute nursing curriculums that are aligned to the practice setting. It is vital that nurse educators should keep themselves at par with the dynamic clinical setting [27]. The mentorship program fits very well to provide nursing faculty with an opportunity to update their clinical skills [28]. Faculty development is helpful to equip faculty staff to recognize students’ needs and develop strategies to support these needs [29].

Findings have reported strategies to build capacity of clinical staff through preceptorship and mentorship. There is evidence of adequate learning gains by students when clinical staff take charge of student learning [30]. Nurses could be experts in their clinical field but might not be able to transfer such expertise to students. Most clinical nurses might feel unprepared and disconnected when handling students. Lack of proper capacity building causes a feeling of lack of support in their role and inadequate preparation for clinical staff. Building capacity of clinical staff through mentorship facilitates appropriate communication and interaction between clinical staff and students, incorporation of both theory and practice. Preceptors facilitate development of students’ practical skills and facilitate professional socialization [31]. Students who are being supported by preceptors have a widened knowledge base and clinical skills [32]. Preceptors develop self-confidence and there is better communication [33]. Maintaining a positive clinical placement that promotes learning through effective role modelling and clinical teaching where students are given the appropriate attention, nurtured and supported ensures adequately prepared graduates.

Msiska, Smith [34] reported that in many clinical settings in Malawi, the clinical learning environment was hostile and oppressive due to negative attitudes of staff towards students. Findings of this study show a considerable effort to improve clinical learning environment. Introduction of ideal teaching model wards was necessary to provide students with positive clinical learning environment. A good clinical learning environment is crucial to develop learning skills, clinical reasoning and develop students as professional nurses. A positive clinical learning environment facilitates acquisition of competencies. Studies have shown that a positive clinical environment is one in which students feel confident, motivated to learn, are respected and recognised [35]. The strategies been implemented are fostering a sense of cooperation and willingness for staff to teach students. Students are given appropriate guide to provide quality care [36, 37].

The Regional Professional Regulatory Framework for Nursing and Midwifery reported that efforts to improve nursing and midwifery tend to ignore regulation [38]. Building capacity of regulatory bodies is paramount to quality check and maintenance of standards in nursing education [24]. Educational institutions and professional nursing organizations need to actively contribute to the development of health policy on regulation [14].

In a poor resource setting like Malawi, simulation with high- fidelity technology is an innovative and effective clinical teaching strategy. Simulation addresses the challenge of increased student enrolment, faculty shortage and limited clinical sites. Weaver [39] and Parker and Myrick [40] have documented multiple benefits of simulation in nursing education. These are an increased learner satisfaction, teamwork, increased self- confidence and promotion of clinical judgement and critical thinking. Richardson, Goldsamt [41] observed that substituting simulation for traditional clinical hours can be a sustainable and educationally sound option to increase faculty capacity. Students tend to have more time for clinical practice when using simulation [42].

### 5.1 Implications for Practice and Education

An effective transformation and scale up of nursing education requires collaboration between academia and practice. Effective transformation of nursing education will need adequate capacity building for both nurse practitioners and nurse educators. A model for transformation would provide appropriate guidance on transformation while giving attention to standards in teaching and learning.

### 5.2 Implication for Research

There is need to evaluate the impact of nursing education strategies on the health system and health outcomes. More research would be required to understand the operationalization of each specific strategies being implemented to improve nursing education in Malawi.

## CONCLUSION

Nursing education remains critical to building a strong health system. Transforming and scaling up of nursing and midwifery improves the quality, quantity and relevance of nursing education. Considering that the healthcare environment is increasingly becoming complex, dynamic with changing demands, transformation of nursing education is needed than before. Improvement in nursing education needs to match these changes if the nurses are to effectively perform and positively impact on patient and care outcomes. Findings of this study have shown that the strategies being implemented to improve nursing education are relevant to closing the gap that exists between health care needs and nursing education. Building capacity of nursing faculty, clinical nurses will ensure that nursing student acquire the necessary knowledge, skills and competencies to provide quality and safe care. Provision of adequate resources and a conducive learning environment strengthens teaching and learning for students.

## Figures and Tables

**Table 1:** Demographic characteristics of respondents.

Variable	%	n
Gender	-	-
Male	85	136
Female	15	24
Age	-	-
Less than 25 years	49	79
26 - 30 years	27	43
31 - 35 years	13	20
36 - 40 years	6	10
More than 41 years	5	8
Cadre	-	-
Nurse Midwife Technician	56	90
Registered Nurse	44	70
Education qualification	-	-
Diploma (NMT)	56	90
Diploma (RN)	29	46
Bachelors degree	11	18
Masters degree	4	6
Area of practice	-	-
Nurse practitioner	76	121
Nurse educator	24	39

**Table 2:** Respondents’ opinion.

-	Mean	SD
Student recruitment	-	-
Proper system in place	1.30	0.77
Implementation properly done	1.82	1.40
Strategies to improve recruitment are in place	4.56	0.68
Standards of recruitment are available	1.06	0.24
Teaching and learning	-	-
There is appropriate collaboration in teaching students	2.07	1.10
Teaching and learning effectively done	1.83	1.06
Strategies are place to improve teaching and learning	1.46	0.76
Standards on teaching and learning in place	1.30	0.64
Clinical practice	-	-
Strategies to improve clinical teaching in place	1.57	0.71
Clinical learning environment is conducive	3.02	1.24
Adequate collaboration in teaching students	2.62	1.32
Standards on clinical teaching in place	1.27	0.50
Transition to practice	-	-
Nurse educators are involved	4.39	0.74
Formal transition program in place	3.61	1.17
Transition currently effectively done	4.06	0.91
Transition to practice guidelines in place	4.79	0.53
Strategies are available to improve transition to practice	4.69	0.54

Note: 160 participants responded to the questionnaires

**Table 3:** Identified stakeholders (Main).

**Stakeholder**	**Main objective **	**Interventions**
ICAP Nursing Education Partnership Initiative (NEPI)	To improve production, quality and relevance of nurses and midwives to address essential population-based needs, including HIV and other life threatening conditions	Teaching and learning infrastructure
-	-	Faculty capacity for designing and delivering relevant, quality education
		Access to quality teaching/learning in the classroom and clinical contexts
		Education management capacities of faculty and support staff
		Innovative models and practices for nursing and midwifery programs
		Evidence-base for strategic, education, management decision-making
		Ability of regional and local organizations and partners to provide technical assistance and capacity building support for nursing and midwifery professional development activities within existing systems
		Leadership, administrative and financial competencies of key faculty, administrative staff and institution
Clinton Health Initiative (CHAI)	To work with governments to address this massive shortage and produce high quality, sustainable healthcare systems.	Health education infrastructure
-	-	Health workforce management necessary to produce high quality, sustainable healthcare systems.
		Funding salaries of additional lecturers
GAIA	To build health systems by addressing human resource shortage	Funding educations of nursing students
-	-	Train health care professionals in updated antiretroviral therapy (ART) protocols, Basic Emergency Obstetric Care, Triage, and improved Clinical Instruction
MCHIP	To strengthen the country's health systems	Capacity building through training of midwives and preceptors in BEnONC
		Mentorship training programs concentrated on pre-service education
International Training & Education Centre for Health (I-TECH)	To support efforts to strengthen the national health system. Drawing on expertise from I-TECH’s global network,	Quality improvement; program assessments; and support for information systems.
-	-	Providing technical assistance for curriculum revision and standardization, monitoring and evaluation,
Norwegian Church AID (NCA)	To strengthen health services in Malawi in order to decrease maternal and child mortality.	College infrastructure development
-	-	Capacity building through training of tutors in research methods, teaching methods,
-	-	Strengthen college management systems
		Provision of teaching and learning resources

**Table 4:** Summary of strategies and targeted area.

**Area of transformation**	**Strategy**	**Targets area**
Curriculum reform	Community based education	Relevance - universal coverage
-	Inter-professional education	Relevance - skills mix
-	Competency based	Quality - competence
Regulation	Accreditation	Quality - control
-	Licensure	Quality - control
-	Guidelines/ frameworks	Relevance/ quality - direction
Faculty development	Advanced programs	Quality/ relevance - capacity
-	Continuous Professional Development	Quality/ relevance - competence
Transformative teaching	Distance learning	Quantity - access
-	Simulation	Quality -
-	e- learning approach	Quantity - access
Infrastructure/ resources	Clinical learning environment	Quantity - access
-	Teaching space	Quantity
